# Evaluation of Real-time PCR for Diagnosis of Post-Kala-azar Dermal Leishmaniasis in Endemic Foci of Bangladesh

**DOI:** 10.1093/ofid/ofy234

**Published:** 2018-09-15

**Authors:** Prakash Ghosh, Md Golam Hasnain, Faria Hossain, Md Anik Ashfaq Khan, Rajashree Chowdhury, Khaledul Faisal, Moshtaq Ahmed Mural, James Baker, Rupen Nath, Debashis Ghosh, Shomik Maruf, Mohammad Sohel Shomik, Rashidul Haque, Greg Matlashewski, Shinjiro Hamano, Malcolm S Duthie, Dinesh Mondal

**Affiliations:** 1 Nutrition and Clinical Service Division, International Centre for Diarrhoeal Disease Research, Bangladesh, Dhaka, Bangladesh; 2 Centre for Clinical Epidemiology and Biostatistics, School of Medicine and Public Health, Faculty of Health and Medicine, The University of Newcastle, New South Wales, Australia; 3 Department of Microbiology and Immunology, McGill University, Montreal, Québec, Canada; 4 Department of Parasitology, Institute of Tropical Medicine, Nagasaki University, Nagasaki, Japan; 5 Infectious Disease Research Institute, Seattle, Washington, USA

**Keywords:** diagnosis, microscopy, post-kala-azar dermal leishmaniasis (PKDL), real-time PCR

## Abstract

**Background:**

Post-kala-azar dermal leishmaniasis (PKDL) is a sequel to visceral leishmaniasis (VL), which is found in VL-endemic countries including Bangladesh. Because of these enigmatic cases, the success of the National Kala-azar Elimination Program is under threat. To date, diagnostic methods for PKDL cases in endemic regions have been limited to clinical examination and rK39 test or microscopy, and a suitable and accurate alternative method is needed. In this study, we investigated the application of real-time polymerase chain reaction (PCR) as a potential method for diagnosis of PKDL in comparison with microscopy.

**Methods:**

Ninety-one suspected macular PKDL cases from Mymensingh district, Bangladesh, were enrolled in the study after diagnosis by clinical examination and an rK39 strip test. All of them responded after completion of the treatment with miltefosine. During enrollment, a skin biopsy was done for each patient, and both microscopy and real-time PCR were performed for detection and quantification of *Leishmania donovan* body (LDB) and LD DNA, respectively.

**Results:**

Real-time PCR detected 83 cases among all suspected PKDL patients, with an encouraging sensitivity of 91.2% (83.4%–96.1%), whereas microscopy showed 50.6% (39.9%–61.2%) sensitivity. Among all suspected PKDL cases, 42 cases were positive in both microscopy and qPCR, whereas 41 cases were detected as positive through qPCR only.

**Conclusions:**

This study provides evidence that real-time PCR is a promising tool for diagnosis of PKDL in endemic regions. In addition to diagnosis, the quantitative ability of this method could be further exploited for after-treatment prognosis and cure assessment of PKDL cases.

Post-kala-azar dermal leishmaniasis (PKDL) is a sequel of visceral leishmaniasis (VL) that often exhibits lesions or hypo-pigmented skin rashes in patients after successful treatment for VL [1]. This cryptic infection is characterized by papular, macular, and/or nodular lesions all over the body, mainly on the face, trunk, legs, arms, and genitals [2]. PKDL cases with various types of lesions are discretely distributed across VL-endemic regions. In Sudan, nodular or papular lesions are prevalent in PKDL patients, with an incidence rate of 51%, whereas the incidence rates for maculopapular, micropapular, and macular lesions are 23%, 17%, and 9%, respectively [3]. In India, the distribution rates of macular and other polymorphic nodular lesions are 23% and 45%, respectively, whereas in Bangladesh the most prevalent form of PKDL is macular [4–6]. Like multiple lesion types in PKDL, the developmental rate of PKDL among treated VL patients varies over the regions. In Sudan, 50%–60% of treated VL patients develop PKDL within weeks to a few months after treatment of VL, and most of the cases are self-healing [2]. In India, this rate is 5%–10% within 2–4 years after treatment, and in Bangladesh, it is 10% within 36 months after completion of VL treatment [5, 7]. Moreover, the manifestation of PKDL without a prior history of VL is not uncommon. According to Ramesh et al., 15%–20% of PKDL patients originate from an asymptomatic form of *Leishmania donovani* infection [8]. Unlike VL, PKDL is not fatal if it remains untreated, but patients with severe cases are often victims of social stigma [9]. Moreover, PKDL cases have been implicated as reservoirs for *leishmania donovani* (LD) parasites [10, 11]. Therefore, elimination of PKDL in addition to VL is critical to achieve the goal of the National Kala-azar Elimination Program (NKEP) [12–14].

Despite having treatment options for PKDL, the success of control measures is complicated due to lack of awareness and poor treatment-seeking behavior of PKDL patients [15]. Moreover, definitive diagnosis of PKDL is problematic because lesions are often confused with vitiligo, leprosy, secondary syphilis, and sarcoidosis [2]. Conventionally, PKDL is diagnosed by detecting amastigotes in slit skin or skin biopsy smear under microscopy. However, the sensitivity of this method is not satisfactory; especially in macular PKDL cases, the sensitivity ranges from 3.6% to 41.6%, whereas other forms of PKDL (papular and nodular) show comparatively higher sensitivity [5, 16]. In last few decades, several serological and immunological methods have been developed to overcome the limitations of microscopy-based diagnostic methods [17]. However, like primary VL diagnosis, immunological methods such as rK39 rapid diagnostic test (RDT) cannot be used as the confirmatory diagnostic tool for PKDL as any *Leishmania* exposed individual develops antileishmanial antibody, and it persists for a long time. Therefore, rK39 RDT is being used as an auxiliary diagnostic test for PKDL [18]. A highly sensitive diagnostic tool would be a significant advance toward solving the problem of PKDL.

Recent studies have shown that various molecular-based diagnostic methods are very sensitive for detecting both VL and PKDL [19–21]. Polymerase chain reaction (PCR)–based methods appear to be more applicable as these can be performed with a broad range of clinical specimens [17]. Several studies have reported an encouraging sensitivity rate of conventional PCR for diagnosis of PKDL using skin specimens [22]. Although conventional PCR has a high sensitivity rate (approximately 94%), a longer performing time and lack of quantification of the number of parasites, which is imperative for monitoring treated patients, limit its potential as a routine diagnostic tool [19, 23–25]. To overcome the pitfalls of conventional PCR-based methods, more recently, quantitative PCR (qPCR) has emerged as an alternative diagnostic tool that addresses many of the limitations of conventional PCR. It requires less amplification time and can accurately quantify parasite load in clinical samples. Several SYBR-green and TaqMan chemistry–based single and multiplex real-time PCR methods have shown significant sensitivity and specificity for the diagnosis of PKDL [20, 26–30]. Recently, we reported 85% sensitivity of a TaqMan-based real-time PCR method in diagnosing PKDL with skin biopsy specimens [21]. However, the study was performed with a limited number of archived samples. To bring this real-time PCR assay closer to practice, we undertook our current study for validation of the assay on a larger scale. We compared the diagnostic efficacy of qPCR with conventional microscopy by assuming the clinical examination along with treatment outcome as the gold standard. Our data indicate the excellent sensitivity of real-time PCR and support the expanded use of this method for diagnosis of PKDL in endemic regions.

## METHODS

### Study Site and Population

This study was conducted in hyperendemic kala-azar areas of Mymensingh district, Bangladesh. Proper consenting procedures were followed during enrollment of the participants. The study was conducted between the period of January 2016 and July 2016, and the design was approved by the International Centre for Diarrhoeal Disease Research Bangladesh (icddr,b) Institutional Review Board. Participants were prescreened through enrollment into a clinical trial (ClinicalTrials.gov Identifier: NCT02193022) examining the efficacy of miltefosine for treatment of child and adolescent PKDL patients. In the study reported here, we enrolled a subset of 91 suspected PKDL patients from the trial. These patients were diagnosed clinically, and all had a history of VL and skin rash and were positive on rK39 RDT. All presented with macular lesions. Each of the PKDL cases was further evaluated by microscopy and qPCR for the purposes of the present study. Among 91 suspected PKDL patients, 83 cases positive by microscopy or qPCR were included in the study. All the patients were treated with, and responded well to, miltefosine, and treatment response was assessed 1 year after completion of the treatment. Excluded cases were referred to the government treatment facility for further management. In addition to the PKDL cases, 86 age- and sex-matched healthy controls with a previous history of kala-azar were enrolled.

### Specimen Collection

Three separate 3-mm skin biopsy samples were collected from each suspected PKDL case. After collection, 2 biopsies were preserved in the NET buffer for DNA extraction, and the other was used for skin smear. From each patient, 3 mL of venous blood was also collected. Due to ethical issues, only blood samples (3 mL) were collected from healthy controls. After collection, samples were maintained in a cold chain and transported to icddr,b. Trained laboratory personnel performed all laboratory investigations at an emerging infection and parasitology laboratory, icddr,b.

### Microscopy of LD Bodies

The skin smear was prepared with the biopsy material immediately after collection and then allowed to air dry. After that, the smear was fixed with a few drops of absulate methanol and again allowed to air dry and then stained with Giemsa stain diluted in 1X PBS buffer (1:10, PH 6.8). After staining, the slide was washed with tap water and allowed to air dry. Two expert microscopists examined the slides to identify the LD bodies. Discrepancies were resolved through further review of the slides. The grading of the LD bodies was achieved according to the World Health Organization 1991 splenic aspiration microscopic report grading system. LD bodies detected in microscopy were graded as grade 0 (0 parasites in 1000 microscopic [100X-Oil immersion objective] fields), grade 1 (1–10 parasites in 1000 fields), grade 2 (1–10 parasites in 100 fields), or grade 3 (1–10 parasites in 10 fields). Further Z-N stained slides were prepared with skin biopsy material to exclude the presence of *Mycobacterium leprae* (leprosy patients).

### DNA Extraction From Clinical Specimen

Blood collected in an EDTA tube was centrifuged at 2200*g* for 20 minutes for separation of the buffy coat. DNA was isolated from 200 μL of buffy coat and skin punch biopsies using a QIA amp DNA tissue & blood mini kit (Qiagen, Hilden, Germany) as per the manufacturer’s instructions. For skin biopsies, the extraction method was improvised to get a high yield of DNA. According to the modified method, skin biopsy materials were kept at 37°C overnight after addition of ATL buffer and protease K. On the following day, the skin materials were homogenized and then incubated at 56°C for 2 hours before further processing according to the manufacturer’s instructions. Extracted DNA samples were stored at –20°C until real-time PCR.

### Detection and Quantification of LD DNA by qPCR

Real-time PCR was performed as originally described by Vallur et al. [31]. Briefly, TaqMan primers and probes targeting a conserved region of *Leishmania* REPL repeats (L42486.1) specific to *L. donovani* and *L. infantum* were synthesized by Applied Biosystems [31] and a 20-μL reaction mix prepared containing a 5-μL template, 10 μL of TaqMan Gene Expression Master Mix (2X), 1 μL of pre-ordered primer-probe mix, and PCR-grade water. Amplification was performed on a Biorad CFX96 iCycler system with the following reaction conditions: 10 minutes at 95°C, followed by 45 cycles of 15 seconds at 95°C and 1 minute at 60°C. To quantify the parasite load of each sample, each run included 1 standard curve with DNA concentrations ranging from 10 000 to 0.1 parasites [21]. Each run also included 1 reaction with molecular-grade water as a negative control. Each DNA sample was evaluated in triplicate. Samples with a cycle threshold (Ct) >40 were considered negative.

### Statistical Analysis

Parametric and nonparametric tests were performed based on the distribution of data. Kappa and McNemar’s test was performed to determine the concordance and discordance between microscopy and qPCR. Standard statistical formulas were followed to determine the sensitivity and specificity of the test with 95% confidence intervals. All statistical analyses were performed using SPSS (version 20.0) and GraphPad Prism (version 7.03). A *P* value <.05 was considered statistically significant.

## RESULTS

**Figure 1.**
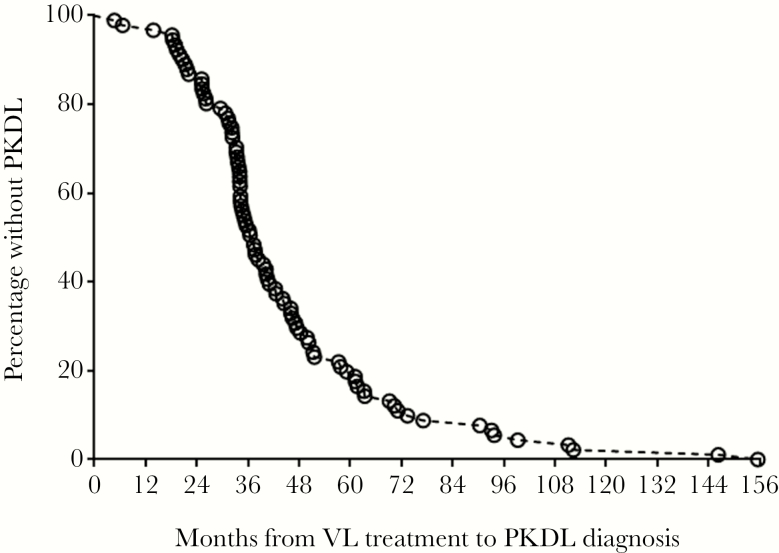
Time gap between diagnosis for post-kala-azar dermal leishmaniasis and treatment for visceral leishmaniasis. Abbreviations: PKDL, post-kala-azar dermal leishmaniasis; VL, visceral leishmaniasis.

An improved understanding of how PKDL develops and how to detect it is required for VL control programs. Among 91 confirmed child and adolescent PKDL patients, 47.3% were male, and the mean age of the cohort (SD) was 129.57 (39.23) months. The clinical examination confirmed all the PKDL patients as macular cases. In addition, the male:female ratio in the control group was similar (0.9:1), where the mean age of control participants (SD) was 129.7 (38) months. The survival analysis indicated that most of the patients (n = 59) developed PKDL within 4 years after VL treatment (Figure 1).

**Figure 2.**
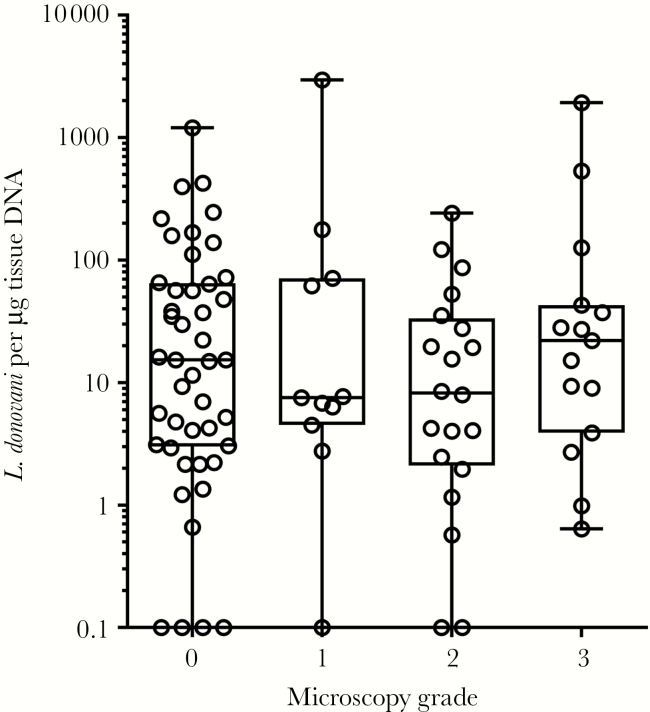
Comparison of parasite loads across the grades.

All the PKDL patients were diagnosed on the basis of clinical exam and clinical history. Through microscopy, 46 patients were confirmed as containing LD bodies in their skin smear (overall sensitivity of 50.55% [39.86%–61.20%]) (Table 1), whereas all were found to be negative for *M. leprae*. Following the World Health Organization grading system for LD bodies, microscopy determined 11 (23.91%) patients as grade 1, 20 (43.48%) as grade 2, and 15 (32.61%) as grade 3. qPCR detected 83 PKDL patients as positive with a sensitivity of 91.21% (83.41%–96.13%) when it was performed with skin biopsy DNA (Table 1), but none was found qPCR positive with buffy coat DNA. We found very poor agreement (Kappa = 0.002) between microscopy and real-time qPCR results, and the methods were extremely discordant (*P* = .000).The overall median parasite load in microscopy-positive cases was quantified (interquartile range [IQR]) at 9.19 (3.61–45.44), with median parasite loads of 7.56 (4.5–71.22), 8.22 (2.09–33.42), and 22.06 (3.9–43.02) per ug tissue DNA, respectively, for grade 1, grade 2, and grade 3 (Figure 2).The median parasite load per ug tissue DNA (IQR) was found 15.3 (2.99–64.7) through qPCR in microscopy-negative samples, further indicating the augmented ability of qPCR to detect LD in skin samples from PKDL patients. The differences in parasite loads quantitated in qPCR across the 4 gradings were not significantly different (*P* = .2457) (Figure 2), and no correlation was found between the parasite load and time between VL treatment and onset of PKDL (*P* = .690). By assuming microscopy or qPCR as the confirmatory test, we found sensitivities of 95% and 52% for qPCR and microscopy, respectively. The high sensitivity of qPCR was provided at an absolute specificity, as all the controls were negative.

**Table 1.** Distribution of PKDL Cases in Accordance to Interval in Years, Along With Diagnostic Outcomes

Interval (VL Treatment to Diagnosis for PKDL), y	No. of PKDL Patients	Microscopy (Grade)			qPCR (positive)
		1	2	3	
0–2	12	1	5	1	12
2–4	52	4	12	8	47
4–6	17	4	1	3	15
6+	10	2	2	3	4
Total	91	46			83
Sensitivity (95% CI), %		50.6 (39.86–61.20)			91.2 (83.41–96.13)

Abbreviations: PKDL, post-kala-azar dermal leishmaniasis; VL, visceral leishmaniasis.

## DISCUSSION

The Kala-azar Elimination Program (KEP), conceived in 2005, has an overreaching goal of eliminating the disease from the Indian subcontinent [32]. The program encompasses multiple approaches, including early diagnosis and complete case management, as well as effective disease surveillance through active and passive case detection. Tremendous success has been made in reducing the VL case rate to <1 per 10 000 population in endemic regions of Bangladesh and Nepal [11]. The success of the attack phase of KEP is prompting the initiation of a consolidation phase to sustain elimination in endemic zones. In a consolidation phase, the proper identification and control of potential sources of infection will receive priority. Given that it is thought to be an important factor in interepidemic cycles, proper diagnosis and treatment of PKDL is a prerequisite for controlling the transmission of *Leishmania* parasites. A recent review by Zijlstra et al. indicated that validation and implementation of molecular tools, including qPCR, are imperative for diagnosis of PKDL [11]. To address this unmet need, we validated a qPCR assay and propose its potential within the diagnostic algorithm for PKDL patients with macular lesions.

Prior reports on the diagnosis of macular PKDL cases has been limited because such cases are quite rare. One study reported 100% sensitivity of qPCR in diagnosing PKDL cases, but only 2 macular cases were included [30]. The qPCR data presented here indicate improved sensitivity for confirming PKDL over our previous study, likely because an additional skin biopsy was taken to perform the assay in the event of an initial negative result [21]. We found biopsies from 78 PKDL patients that were positive in the first round of testing, but this increased to a total of 83 patients when an alternative biopsy specimen was evaluated. This observation infers that collection of multiple skin biopsies from each patient enhances the sensitivity of qPCR for macular PKDL cases. Two studies performed previously in Bangladesh reported the sensitivities of PCR in diagnosing macular PKDL cases at 42.9% and 93.2%, respectively [5, 6]. Further studies performed by Verma et al. reported the sensitivities for PCR and qPCR at 87.5% and 75%, respectively, in diagnosing macular PKDL cases [16, 33]. The discrepancy among the studies might be attributed to different geography and target genes used for the PCR. Further, the comparatively lower sensitivity of qPCR relative to other studies from Bihar, India, can be explained by differential presentation (macular vs papular or nodular, and the paucity of the parasite in macular lesions). During PKDL, *L. donovani* parasites reside in the skin, and it was therefore not surprising that parasites were not detected in peripheral buffy coats from any of the PKDL patients. However, this does contrast with a previous study that found 50% of LD DNA to be positive in peripheral buffy coat through conventional PCR [5]. It is difficult to explain this anomaly, although a previous study on cutaneous leishmaniasis suggests that the invasive characteristic of the *Leishmania* parasite can allow *L. major* to escape the skin and circulate through blood [34]. It is possible that *L. donovani* has a very brief transitory stage and, given that our samples were collected after the clinical emergence of PKDL, we may have missed this period. Prospective studies are required to fully address this possibility.

Earlier studies have revealed that, based on the type of PKDL lesion, sensitivity of microscopy is extremely variable (4%–71%) [7, 16]. Several studies reported an improved sensitivity of microscopy-based methods, including imprint microscopy and immunohistochemistry, over conventional skin smear microscopy [16, 35]. Considering only macular cases, our study provided a higher sensitivity in detecting LDB in skin smear by microscopy than has previously been observed. It is also notable that, for the first time, we were able to grade the parasite load in microscopy with a large number (n = 91) of macular PKDL patients.

**Figure 3.**
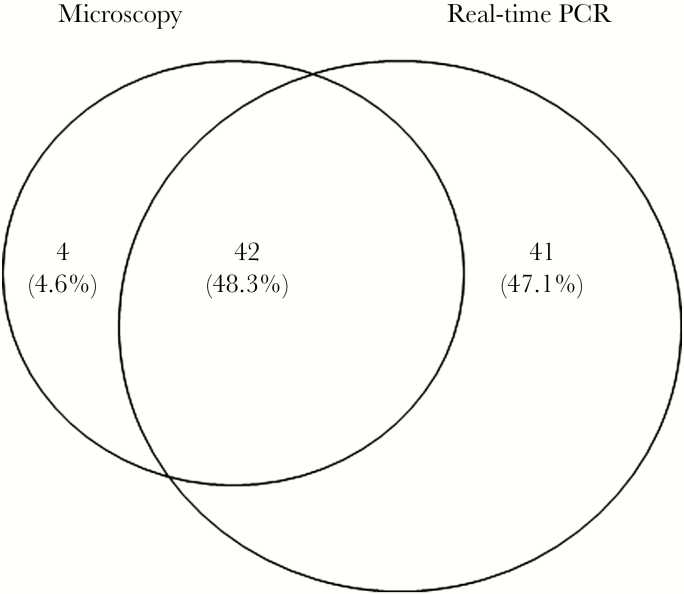
Distribution of post-kala-azar dermal leishmaniasis patients diagnosed through microscopy and/or real-time polymerase chain reaction in a Venn diagram. Abbreviation: PCR, polymerase chain reaction.

Only a weak agreement was observed when comparing microscopy and qPCR for diagnosis of PKDL. In total, 42 cases were positive in both microscopy and qPCR, whereas 41 cases were detected as positive through qPCR only (Figure 3). Surprisingly, 4 cases were found to be positive in microscopy but negative in qPCR (Figure 3). The lack of a statistically significant difference in qPCR-assigned median parasite loads between microscopy-positive and -negative patients nullified attempts to correlate microscopy and qPCR data. Further, even when stratified by microscopy grades 1, 2, and 3, the median parasite loads found in qPCR, we did not observe a strong correlation. We observed large variability in parasite abundance in macular lesions across the endemic regions. Thus, these discrepancies could be attributed to the use of different biopsies for each method and highlight the variability of *Leishmania* burdens between lesions.

Survival analysis of the PKDL patients in our study determined a median time to develop lesions after VL treatment of 38 months, similar to the findings of 2 other studies [5, 36]. The time to PKDL development did not correlate with the parasite load in the skin. According to most reports, the mean parasite load in the macular lesion is approximately 50 per ug tissue DNA, and in our previous study, we found a mean parasite load of 295.46 per ug tissue DNA [21]. Recently, Srija Moulik et al. reported a median parasite load of 3665 per ug tissue DNA, where all (n = 91) of the patients had macular lesions [3]. In the current study, the median parasite load was much lower, at 14.91 (3.05–56.5) per ug tissue DNA, but parasites were still readily detectable [30, 33, 37]. Although the real-time PCR methods between these studies are different, the large discrepancies in parasite load raise questions of the heterogeneous infectivity of macular PKDL cases and their discrete geographical distribution. In the Indian subcontinent, given its multifarious advantages, rK39 RDT is being used for diagnosis of both VL and PKDL cases. In Bangladesh, national guidelines for PKDL diagnosis are that rK39 RDT serves as an ancillary confirmatory test. As expected, in agreement with the previous studies performed by Mondal et al. and Verma et al. [5, 33], all the PKDL patients in this study were positive with rK39 RDT.

The major limitation of this study is that we performed PCR and microscopy with different biopsies. As the distribution of the parasite in skin rashes or lesions is discrete in PKDL, the possibility of over- or underestimation of the efficiency of any of the methods arises. For a robust comparison between methods, further methodological improvisation is required to conduct both microscopy and PCR with the same sample. The need to perform skin biopsy, which is invasive and requires suturing, causes discomfort and limits the number of people who agree to this invasive procedure for diagnostic purposes. Recently, several studies reported the promising diagnostic efficacy of the less invasive slit skin, microbiopsy, and fine needle biopsy methods [24, 33, 38, 39]. Further validation is needed before these methods can be used for PKDL patients.

Because of variable sensitivities and the significant influences of experience of the individual making the smear, the quality of the smear, the reagents used, and the long execution time, the microscopy-based method is poorly adopted for PKDL diagnosis even in tertiary care or referral centers/hospitals in endemic countries. In contrast, our prospective study provides important insight into the applicability of qPCR for diagnosis of PKDL cases with macular lesions, indicating that it is more reproducible and repeatable for the detection of LD DNA. Further, the specific nature of the assay could differentiate PKDL cases from confounding diseases such as leprosy and ensure prompt and appropriate treatment [40]. For routine use, our data indicate that the collection of multiple skin samples can also enhance sensitivity. Our data will help craft effective diagnostic algorithms in regions where macular PKDL cases are prevalent. In endemic foci with tertiary health facilities, this quantitative pPCR method could readily be used to ensure the proper diagnosis, treatment, and monitoring of PKDL cases.
